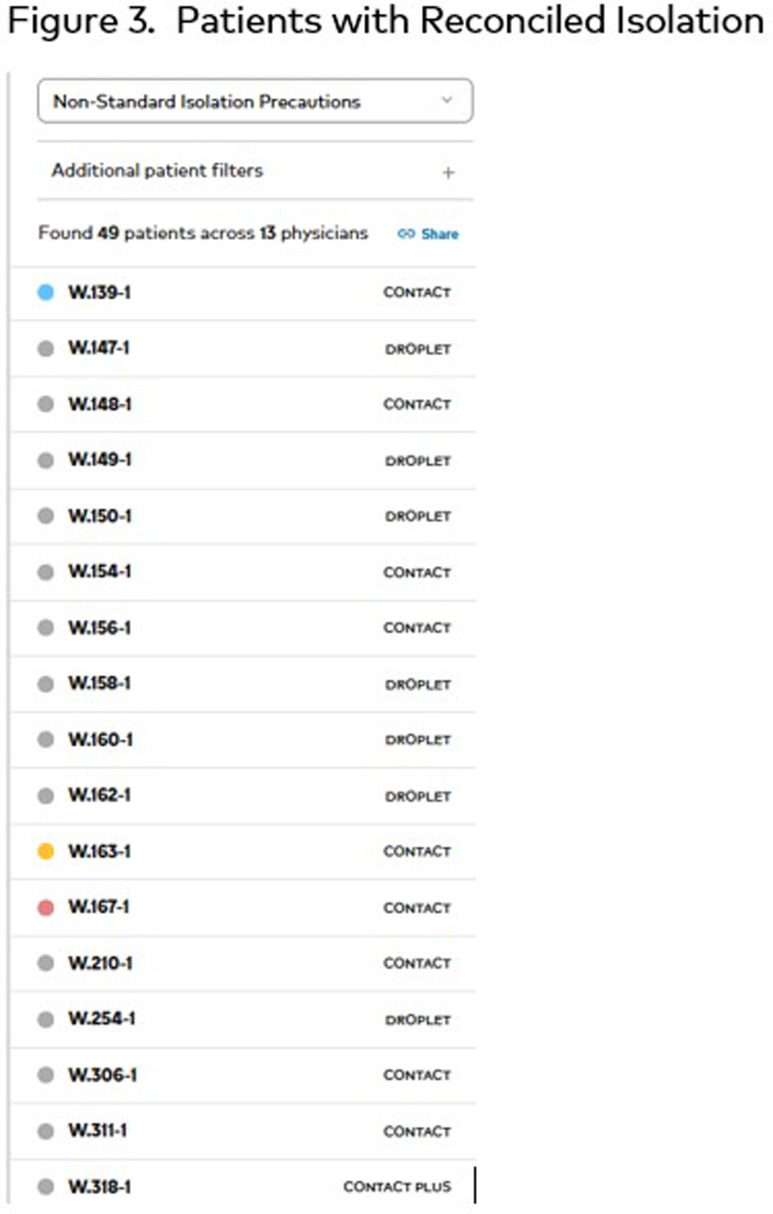# 304 Hospital-onset influenza and RSV: Impact of Infection Prevention Efforts During the COVID-19 Pandemic

**DOI:** 10.1017/ash.2026.10658

**Published:** 2026-06-23

**Authors:** Julia Moody, Michelle Clark, Grayson Ruhl, Suzy Denno, Alex Stephens, Meghna Sathe, Keetha Kratzer

**Affiliations:** 1 HCA Healthcare; 2 HCA; 3 HCA Healthcare corporate ofice

## Abstract

**Background:** A foundational daily activity for infection preventionists (IP) is reconciling nursing isolation documentation to surveillance system current and historical isolation markers. Inefficiencies and isolation delays occur when: (i) electronic healthcare records [EHR] and IP surveillance tools are separate applications requiring manual person dependent resolution; (ii) EHR has logic limited isolation alerts or lack patient isolation history markers to activate on readmission; and (iii) IP staffing when limited to weekdays. Outcomes result in potential communicable disease exposures and added resources for bed reassignment and room cleaning of semi-private rooms. Surveys identified up to 25% of patients require a change in patient isolation status in an average 250-300 bed acute care hospital. **Methods:** EHR nursing isolation was aggregated to isolation alerts from IP third party surveillance software into a technology enabling advanced analytics platform in use for daily device rounds. Real time data overlays updated for admitted patients including pending ER hold admissions. Nursing and IP developed rules defining match agreement and review isolation conditions, visual displays and patient specific information. A pilot proof of concept was tested in 11 acute care hospitals ranging in size from 100 to 740 beds displaying both full facility and unit specific views. High performing nursing units operationalized the tool at the beginning of each shift during daily patient safety rounds including weekends and holidays. **Results:** Tool visuals displayed a patient list to prioritize review of isolation status (Figure 1), patient specific details of mismatch (Figure 2) and reconciled isolation (Figure 3). The average resolution within 24h was 86% (range 79-95%). Charge nurse adoption improved time to isolation, reduced reliance on IP manual processes, improved first time bed assignments and guided nursing patient care assignments. The tool solved for lab “new results” alert gaps to caregivers for MRSA, Candida auris, etc. and provided timely information to care team members unable to access EHR isolation status. Feedback was overwhelmingly positive from users. Short term limitations to reconciliation occurred for patients with clinical conditions warranting isolation pending diagnostic test results (i.e. C. difficile). **Conclusion:** The technology enabler tool brings together various data streams into a single view for patient care teams and leadership to prioritize activities. The tool enabled shared responsibility between nursing and IP for reconciling isolation status, improved communications, driving operational efficiencies and improving safety.

**Figure:**
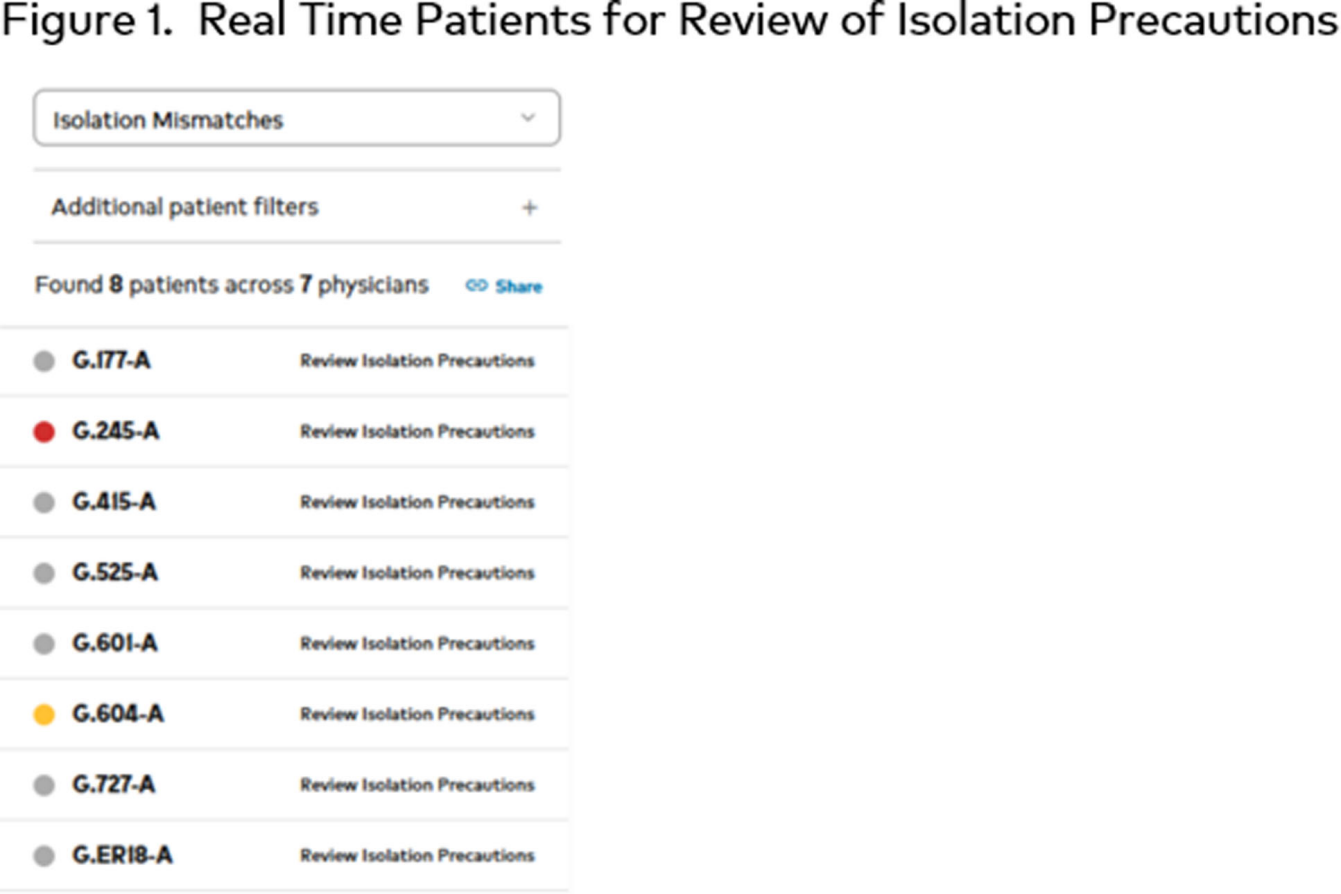

**Figure:**
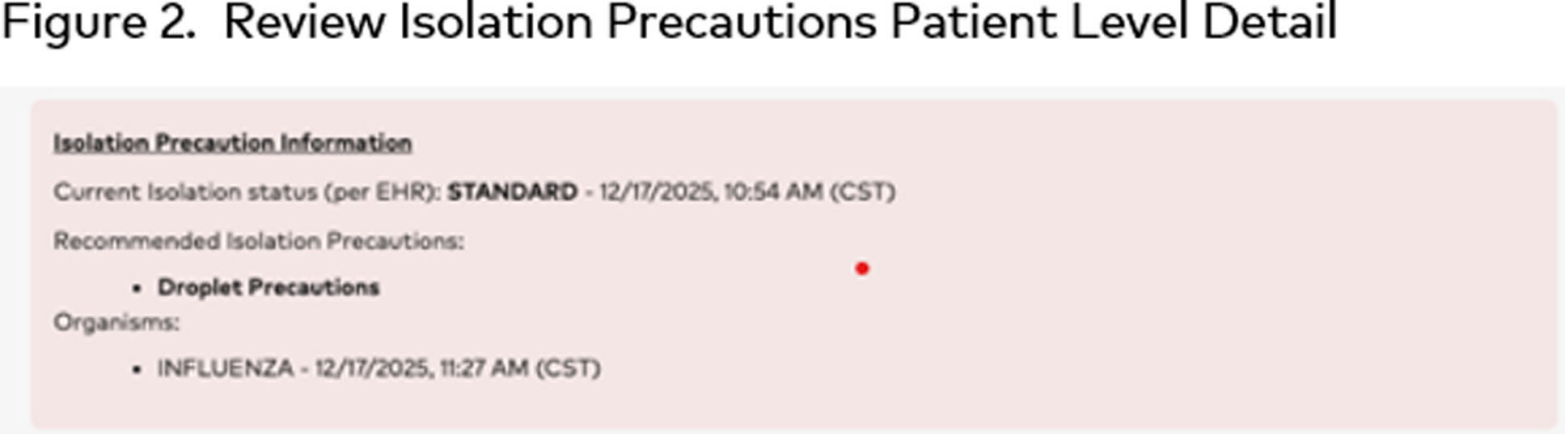

**Figure:**